# A Validation Study on eGFR Equations in Chinese Patients With Diabetic or Non-diabetic CKD

**DOI:** 10.3389/fendo.2019.00581

**Published:** 2019-08-26

**Authors:** Danshu Xie, Hao Shi, Jingyuan Xie, Ying Ding, Wen Zhang, Liyan Ni, Yifan Wu, Yimin Lu, Bing Chen, Hongrui Wang, Hong Ren, Weiming Wang, Na Liu, Nan Chen

**Affiliations:** ^1^Department of Nephrology, Ruijin Hospital, Shanghai Jiao Tong University School of Medicine, Shanghai, China; ^2^Biomedical and Health Informatics, University of Washington, Seattle, WA, United States; ^3^University of Lausanne, Lausanne, Switzerland; ^4^Department of Pharmacy, Ruijin Hospital, Shanghai Jiao Tong University School of Medicine, Shanghai, China; ^5^Department of Nephrology, Shanghai East Hospital, Tongji University School of Medicine, Shanghai, China

**Keywords:** diabetes, chronic kidney disease (CKD), diabetic kidney disease, glomerular filtration rate, CKD-EPI

## Abstract

**Aims:** It remains controversial to choose the optimal equation to estimate glomerular filtration rate (GFR) in chronic kidney disease (CKD) patients with diabetes.

**Materials and Methods:** Two hundred and fifteen diabetic CKD patients and 192 non-diabetic CKD patients were enrolled in this study. Iohexol GFR, serum creatinine (SCr), and Cystatin C(CysC) were measured simultaneously for each patient. SCr- and CysC-based estimated GFR (eGFR) were calculated through eight equations, including three CKD-EPI equations, Revised Lund-Malmö study equation (RLM), CAPA equation, and three Full Age Spectrum (FAS) equations. Bias, precision, and accuracy were compared among eGFR equations with iohexol-GFR serving as measured GFR (mGFR). Independent predictive factors of accuracy were explored using multivariate logistic regression analysis.

**Results:** In the diabetic group, CKD-EPI_SCr−CysC_ showed the best performance among three CKD-EPI equations (interquartile range of 13.88 ml/min/1.73 m^2^ and 30% accuracy of 72.56%). Compared to CKD-EPI_SCr−CysC_, the other five equations did not significantly improve the performance of GFR estimates. Mostly, eGFR equations were less accurate in diabetic group than in non-diabetic group. Significant differences were found in different mGFR range (*P* < 0.001). The multivariate logistic regression analysis identified that BMI, mGFR, and diabetic kidney disease (DKD) status were independent predictors of accuracy of three equations in diabetic group. HbA1c was a predictor of accuracy of CKD-EPI_SCr_ and CKD-EPI_CysC_ in diabetic group.

**Conclusions:** This study showed that eGFR equations were less accurate in the diabetic group than in the non-diabetic group. CKD-EPI_Scr−CysC_ had the best performance among CKD-EPI equations in Chinese diabetic CKD patients. The other five equations did not significantly improve the performance of GFR estimates. BMI, mGFR, DKD status, and HbA1c were independent factors associated with accuracy in eGFR equations.

## Introduction

The estimated overall prevalence of type 2 diabetes in China was 10.9% in 2013 according to a national survey (1). This may result in a proportional increase of chronic kidney disease (CKD) related to diabetes mellitus (2). Indeed, both reduced kidney function and albuminuria are essential not only for the diagnosis of diabetic kidney disease, but also for the prognosis of cardiovascular disease and all-cause mortality (3, 4). Albuminuria could be easily evaluated with simple urine collection. However, direct measurement of glomerular filtration rate (GFR) could be invasive and cumbersome, making it not suitable for day-to-day clinical practice. Thus, a number of equations have been developed to estimate GFR (5–7) and it is of great importance to accurately calculate eGFR. Most of the existing equations were built with serum creatinine (SCr) and serum Cystatin C (CysC). They were mainly developed from studies in CKD populations. Equations based on SCr and CysC, were developed by CKD-EPI (Chronic Kidney Disease Epidemiology Collaboration) group (7, 8). CKD-EPI_SCr_ and CKD-EPI_SCr−CysC_ were recommended by KDIGO in 2012 (9). Later, more equations were developed and were reported for better performance across different age groups, including Revised Lund-Malmö study equation (RLM) (10), CAPA equation (11) and three Full Age Spectrum (FAS) equations (12, 13). However, application of these eGFR equations in diabetic patients remains controversial due to affected levels of SCr and CysC in diabetic status (14).

In this article, we explored which eGFR equations based on filtration markers–SCr, CysC, alone or combined, would better represent GFR with less bias and more accuracy in Chinese diabetic CKD patients. We further explored the factors predicting the accuracy of eGFR.

## Materials and Methods

### Participants

Two hundred and fifteen diabetic patients with chronic kidney diseases (CKD) were recruited from either Ruijin Hospital, Shanghai Jiao Tong University School of Medicine (*n* = 195) or Shanghai East Hospital (*n* = 20). One hundred and ninety two non-diabetic patients with CKD were enrolled at Ruijin Hospital, Shanghai Jiao Tong University School of Medicine. The study period was from December 2013 to December 2016. Patients with CKD and previously or newly diagnosed type 2 diabetes mellitus were enrolled in the experimental group while non-diabetic CKD patients were recruited as a control group. The diagnosis of DKD (diabetic kidney disease) was made through the consensus of at least two senior physicians on the basis of clinical characteristics of DKD, such as diabetes duration and presence of diabetic retinopathy. Other causes of kidney disease were considered if there were atypical features of DKD. Those include sudden onset of low eGFR or rapidly decreasing eGFR, an abrupt increase in albuminuria or development of nephrotic or nephritic syndrome, refractory hypertension, signs of another systemic disease, and >30% eGFR decline within 2–3 months of initiation of a renin-angiotension system inhibitor (15, 16). The exclusion criteria were: (1) patients younger than 18 years old; (2) dehydration or fluid overload including congestive heart failure and severely uncontrolled edema; (3)chronic patients on maintenance hemodialysis or peritoneal dialysis or patients receiving dialysis within the past 3 months; (4) patients diagnosed with acute kidney injury (AKI); (5) patients allergic to iodine or with abnormal thyroid function; (6) patients pregnant or with malignancy; (7) patients on medications which can influence the serum creatinine level (e.g., cimetidine). This study was approved by the institutional review board (IRB) of Ruijin Hospital. Informed consents were signed by patients. Patient's characteristics were collected including gender, age, height, weight, diabetes status, hypertension status, and diagnoses at discharge.

### Samples Taken Procedure

Patients had a light breakfast on the day of blood draw. Five milliliter of Iohexol, namely Omnipaque (300 mg iodine/mL, GE Healthcare, Shanghai, China) was administered. Syringes were weighed to an accuracy of 0.001 g before and after injection of iohexol. The dose of iohexol was calculated by multiplying the absolute difference in syringe weight by the concentration of iohexol (647 mg/mL) and then the result being divided by the density of iohexol (1.345 g/mL).

Iohexol weight (mg) = difference of syringes weight (g)^*^ 647 (mg/mL)/1.345 (g/mL).

**Figure 1:**
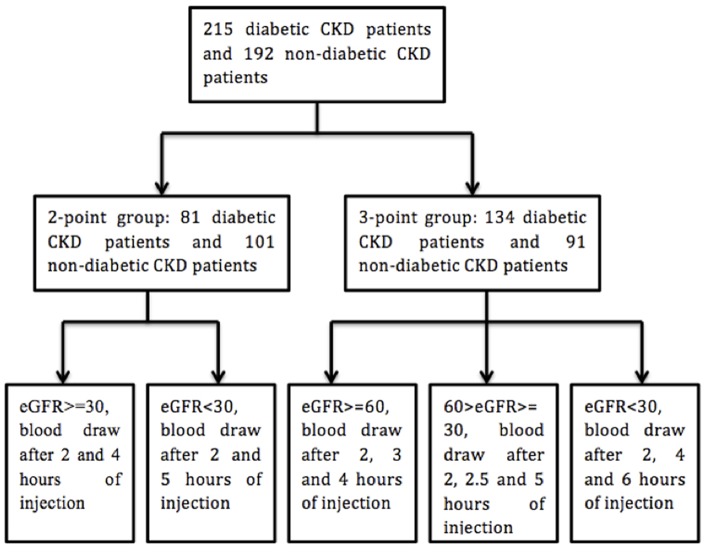
Flow chart of blood draw procedure. Unit of eGFR: ml/min/1.73 m^2^.

All the procedure was done at room temperature. Blood samples were drawn before and after the intravenous injection of iohexol. Phlebotomy site was different from that for intravenous injection. Two kinds of protocols were utilized for the phlebotomy (Figure 1) (17). In our study, the calculated GFR with 2 or 3 times of blood draw after the injection of iohexol (2 or 3 points) showed high consistency (*R*^2^ > 0.98) (For details, please see the Supplemental Figure 1).

### Measurement and Calibration

All the samples were tested in the kidney department's and central laboratories at Ruijin Hospital. We measured SCr by enzymatic method (Beckman Coulter AU5800, KEHUA kit, CV 2.16% at 97.1 μmol/L, 1.65% at 616.6 μmol/L), traceable to isotope-dilution mass spectrometry (IDMS). CysC was measured by Immuno-nephelometry (Particle enhanced, Beckman CoulterAU5800, Sysmex kit, CV 5.71% at 0.49 mg/L, 4.13% at 1.96 mg/L).

Measured GFR (mGFR) was determined by iohexol GFR. With measurement of iohexol by high performance liquid chromatography, iohexol GFR was calculated with the slope-intercept technique and corrected with Brøchner-Mortensen equation (18). BSA was normalized to 1.73 m^2^ with Dubois method (19).

### Calculation of eGFR

The eGFR values were calculated with eight eGFR equations. These included CKD-EPI_SCr_ (7), CKD-EPI_CysC_ (8), and CKD-EPI_SCr−CysC_ (8), RLM, CAPA equation, and three FAS equations (FAS_SCr_, FAS_CysC_, and FAS_SCr−CysC_) (Supplemental Table 1).

### Statistical Analysis

The non-parametric and chi-square tests were used to compare differences between groups in non-normal and continuous variables with uneven variances. Other continuous variables were compared by independent *T*-test. *P* < 0.05 was considered statistically significant when comparing the clinical characteristics. Bland-Altman plots were applied in analyzing the agreement between the mGFR and eGFR. In addition, the performance of eGFR equations was classified as describing bias, precision and accuracy (20). Bias was measured as the absolute or non-absolute median of the difference between eGFR and mGFR. Precision was determined as the interquartile range (IQR) for difference (20). Accuracy was defined as the percentage of differences between eGFR and mGFR within 30% (P30) or 10% (P10) of mGFR. Confidence intervals (CIs) for the metrics were calculated by means of bootstrap methods (1,000 bootstraps) (8). When the 95% CI of non-absolute bias includes zero, the equation is considered unbiased. The differences of absolute bias, precision, and accuracy were compared in 2 steps between equations. First, we compared CKD-EPI_CysC_ and CKD-EPI_SCr−CysC_ with CKD-EPI_SCr_, respectively. Second, the other five equations were compared to CKD-EPI_SCr−CysC_. Absolute bias among equations were compared with paired *t*-test. Precision of equations was compared using the variance ratio test (*F*-test). The difference of accuracy (P30 and P10) was calculated using the exact McNemar test. As we made 7 pairwise comparisons, we used *P* < 0.05/7 = 0.0071 to claim significant difference, according to Bonferroni correction. The performance of each equation was then compared to itself between diabetic and non-diabetic CKD group.

The cross-sectional associations of variables and accuracy were calculated using the chi-square test to find out whether there were significant differences among groups. The associations were further assessed using logistic regression analysis. All the statistical analyses were conducted with IBM SPSS Statistics (version 20.0, Chicago, IL, USA) and R (version 3.3.1, R Development Core Team).

## Results

### Participant Characteristics

In the diabetic CKD group (*N* = 215), the average age was 58.3 ± 11.1 years and 68.4% (*N* = 147) were males. In the non-diabetic CKD group (*N* = 192), the average age was 56.8 ± 13.0 and 58.9% (*N* = 113) were males. The mean mGFR were 49.20 ± 29.71 and 51.54 ± 33.31 (ml/min/1.73 m^2^) for the diabetic CKD and non-diabetic CKD groups, respectively. There were no significant differences for age, gender, corticosteroids use, mGFR, SCr, and CysC level between the two groups (Table 1). The median level of CRP and ESR in diabetic CKD group were 0.44 (*N* = 183, 95% CI: 0.36, 0.56) mg/L and 22.5 (*N* = 176, 95% CI: 18, 27) mm/h. In non-diabetic CKD group, the median level of CRP and ESR were 0.34 (*N* = 181, 95% CI: 0.28, 0.43) mg/L and 18 (*N* = 177, 95% CI: 17, 22) mm/h, respectively.

**Table 1:** Clinical characteristics of CKD with DM and CKD without DM group.

	**Total (*n* = 407)**	**CKD with DM (*n* = 215)**	**CKD without DM (*n* = 192)**	***P***
Age	57.6 (12.0)	58.3 (11.1)	56.8 (13.0)	0.22
Male sex	260 (63.9%)	147 (68.4%)	113 (58.9%)	0.05
BMI, kg/m^2^	25.4 (3.7)	25.8 (3.8)	25.0 (3.6)	0.04
BMI ≥ 28	92 (22.6%)	58 (27.0)	34 (17.7)	0.03
Corticosteroids	53 (13.0%)	23 (10.7%)	30 (15.6%)	0.14
mGFR, mL/min/1.73 m^2^	50.30 (31.43)	49.20 (29.71)	51.54 (33.31)	0.45
Creatinine, mg/dL	2.19 (1.74)	2.22 (1.72)	2.15 (1.76)	0.70
Cystatin C, mg/L	2.22 (1.28)	2.24 (1.30)	2.20 (1.26)	0.77

*CKD, chronic kidney disease; DM, diabetes mellitus; BMI, body mass index. Data were showed as mean (SD). P <0.05 was considered statistically significant*.

### Test Results

#### Bias, Precision, and Accuracy

The bias was represented by median difference and absolute median difference (ml/min/1.73 m^2^) between eGFR and mGFR. The precision was represented by interquartile range (ml/min/1.73 m^2^). The accuracy was represented by differences between eGFR and mGFR. The differences within 30 and 10% of mGFR were showed as P30 and P10, respectively. The greater value means a higher accuracy. Kidney disease outcomes quality initiative (K/DOQI) recommended that accuracy of the eGFR equations should reach 70% and above for P30 (21). Overall, eGFR equations have lower bias, higher IQR, and higher P30/P10 in the non-diabetic group compared to the diabetic group (Table 2 and Supplemental Table 2).

**Table 2:** Bias, precision, and accuracy of different equations in CKD patients with DM.

	**Bias**		**Precision**		**Accuracy**	
	**MD**	**AMD**	**IR**	**P30**	**P10**
CKD-EPI_SCr_	−1.92 (−3.96, 0.11)^a^	8.18 (6.64, 9.38)	16.53 (14.19, 18.62)	72.09 (66.15, 78.16)	25.12 (18.47, 29.91)
CKD-EPI_CysC_	−7.93 (−10.12, −6.38)	9.91 (8.05, 11.51)^*^	15.62 (13.03, 17.71)^*^	60.00 (51.37, 64.94)^*^	20.00 (13.47, 23.92)
CKD-EPI_SCr−CysC_	−6.17 (−8.02, −4.62)	8.46 (6.64, 9.10)	13.88 (11.34, 15.73)^*^	72.56 (66.48, 78.36)	29.30 (21.80, 33.94)
RLM	−3.83 (−4.70, −2.63)	7.77 (6.04, 9.39)	15.57 (12.82, 18.49)	73.49 (67.54, 79.33)	26.05 (18.76, 30.12)
CAPA	−7.54 (−10.09, −6.40)	9.53 (7.92, 11.28)^#^	16.09 (13.07, 18.46)	56.28 (49.17, 62.46)^#^	20.93 (15.27, 25.64)
FAS_SCr_	0.03 (−2.19, 1.34)^a^	6.78 (6.17, 8.20)	13.64 (10.87, 15.68)^#^	75.81 (67.44, 79.35)	29.77 (22.21, 34.75)
FAS_CysC_	−4.49 (−6.64, −1.96)	8.66 (7.34, 10.32)^#^	16.60 (13.17, 18.80)	65.12 (58.14, 71.13)	25.12 (16.73, 27.91)
FAS_SCr−CysC_	−2.41 (−4.40, −1.24)	6.66 (6.07, 8.30)	14.77 (12.13, 16.58)	76.74 (69.87, 81.65)	32.09 (25.69, 37.69)

*MD, median difference; AMD, absolute median difference; IR, interquartile range. Data were showed as value (95% confidence interval, 95% CI). CIs for the metrics were calculated by means of bootstrap methods (1,000 bootstraps). Unbiased results are^a^*.

* *P <0.0071, compared to CKD-EPI_SCr_*.

# *P <0.0071, compared to CKD-EPI_SCr−CysC_. RLM, Revised Lund-Malmö equation; FAS, Full Age Spectrum equation*.

In the subgroup of diabetic CKD patients, bias, accuracy and precision were also compared among the eGFR equations (Table 2). Median difference was unbiased for 2 creatine-based equations, CKD-EPI_SCr_ and FAS_SCr_. Meanwhile, both of them had equal absolute magnitude compared to CKD-EPI_SCr−CysC_ (8.18, 6.78 vs. 8.46 ml/min/1.73 m^2^).

Interquartile range was smaller in CKD-EPI_SCr−CysC_ than CKD-EPI_SCr_ (13.88 vs. 16.53 ml/min/1.73 m^2^). Though the *F*-test showed CKD-EPI_SCr−CysC_ had significantly different precision compared to FAS_SCr_, they had similar interquartile range (13.88 vs. 13.64). The P30 of CKD-EPI_SCr_, CKD-EPI_SCr−CysC_, RLM, FAS_SCr_, and FAS_SCr−CysC_ equation were 72.09, 72.56, 73.49, 75.81, and 76.74%, respectively. They all met the K/DOQI criteria. There was no significant difference among equations in P10.

#### Bland-Altman of Each eGFR Equation in CKD With Diabetic Patients

**Figure 2:**
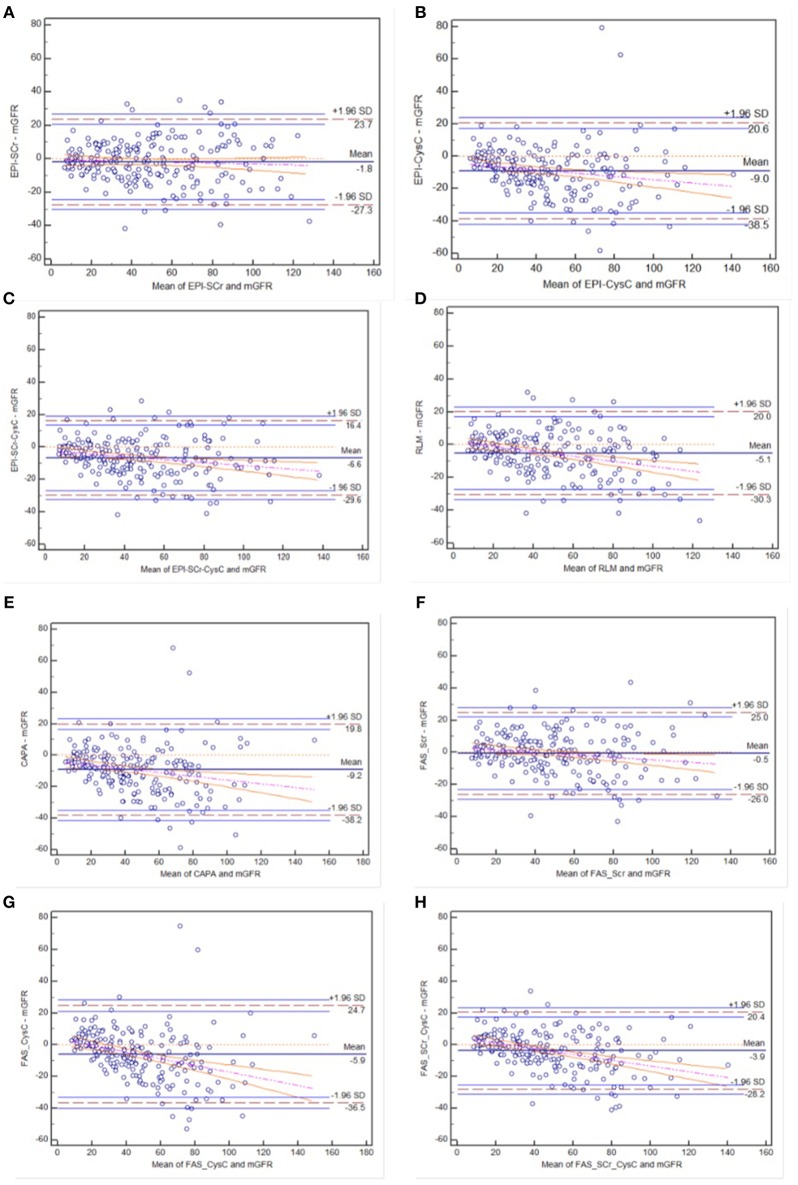
Bland-Altman plots of eight eGFR equations compared to mGFR in diabetic CKD group: **(A)** CKD-EPI-SCr; **(B)** CKD-EPI-CysC; **(C)** CKD-EPI-SCr-CysC; **(D)** RLM; **(E)** CAPA; **(F)** FAS-SCr; **(G)** FAS-CysC; and **(H)** FAS-SCr-CysC. Full line, mean difference between two methods; dashed line, ±1.96 SD difference against mean; the regression line of differences and the 95% confidence intervals are presented.

In the diabetic CKD group, Bland-Altman was plotted with the mean value of eGFR and mGFR as abscissa and the differences between eGFR and mGFR as ordinate (see Figure 2). Among these equations, CKD-EPI_SCr_ and FAS_SCr_ showed overall consistency with mGFR. Among the individuals with higher GFR, the rest equations underestimated GFR.

#### Variables Related to Accuracy of eGFR

In the diabetic CKD group, 180 of 215 patients with HbA1c available within 7 days after GFR measurement were included for the analysis (Table 3). We calculated the difference between eGFR and mGFR. D was defined as the ratio of the absolute difference to mGFR. The eGFR equations with D value <30% were thought to be accurate. The cross-sectional associations of variables and accuracy of the equations were calculated by chi-square test (Supplemental Table 3). Among the six variables (age, gender, BMI, mGFR, DKD status, and HbA1c), mGFR was found being related to the accuracy of CKD-EPI_SCr_ (*P* < 0.001). Also, mGFR and presence of diabetic nephropathy were factors influencing the accuracy of CKD-EPI_SCr−CysC_, respectively. Further associations were evaluated using multivariate logistic regression analysis (Table 4). The results of the analysis indicate that BMI, mGFR and diabetic kidney disease (DKD) status were independent predictors of accuracy of three equations in diabetic group. HbA1c was a predictor of accuracy of CKD-EPI_SCr_ and CKD-EPI_CysC_ in diabetic group.

**Table 3:** Clinical characteristics of 180 patients.

	**CKD with DM (*n* = 180)**
Age, years	58.5 (11.0)
Male sex (%)	120 (66.7%)
BMI, kg/m^2^	26.0 (4.0)
mGFR, mL/min/1.73 m^2^	50.34 (30.19)
Creatinine, mg/dL	2.16 (1.68)
Cystatin C, mg/L	2.22 (1.33)
HbA1c, %	6.9 (1.2)
HbA1c, mmol/mol	51.6 (13.2)

*BMI, body mass index*.

**Table 4:** Logistic regression analysis of variables.

**Variables**	**CKD-EPI**_****Scr****_		**CKD-EPI**_****CysC****_		**CKD-EPI**_****SCr-CysC****_	
	**OR (95% CI)**	***P***	**OR (95% CI)**	***P***	**OR (95% CI)**	***P***
BMI	0.937 (0.926–0.948)	<0.001	0.945 (0.935–0.955)	<0.001	0.899 (0.889–0.091)	<0.001
mGFR						<0.001
≥60	8.791 (7.681–10.061)	<0.001	1.375 (1.226–1.542)	<0.001	3.468 (3.040–3.958)	<0.001
30–60	5.108 (4.559–5.723)	<0.001	0.788 (0.711–0.872)	<0.001	1.673 (1.501–1.865)	<0.001
<30	1	–	1	–	1	–
DKD status						<0.001
Non-DKD	0.609 (0.549–0.677)	<0.001	1.698 (1.549–1.862)	<0.001	1.331 (1.205–1.470)	<0.001
DKD	1	–	1	–	1	–
HbA1c	0.841 (0.807–0.876)	<0.001	1.183 (1.140–1.228)	<0.001	0.943 (0.915–0.933)	0.054

*DKD, diabetic kidney disease; mGFR, measured glomerular filtration rate; OR, odds ratio; 95% CI, 95% confidence interval*.

## Discussion

There are several eGFR equations widely accepted and applied in clinical practice. In this study, we compared three CKD-EPI equations, RLM equation, CAPA equation and three FAS equations in CKD patients with and without diabetes. Our study has shed a light on the precision, bias and accuracy of these equations in the Chinese Han population.

Our study showed that compared to the non-diabetic group, the bias and IQR were higher and P30 and P10 of eGFR equations were lower in diabetic group, which means the eGFR equations were more biased, less accurate and precise in the diabetic group. Previous studies showed that eGFR equations underestimated GFR in diabetic patients with preserved GFR (22, 23). Liu et al. also showed that eGFR equations, re-expressed 4-variable MDRD equation, the CKD-EPI equation and the Asian modified CKD-EPI equation showed more bias, less precision and accuracy in diabetic patients than non-diabetic patients in Chinese population (24). Our results showed consistency with Liu's research.

For eGFR equations with SCr and/or CysC, different performance between diabetic and non-diabetic CKD patients may be determined by several reasons. Firstly, the enzymatic method is better than the Jaffé method in detection for SCr but also performs worse in hyperglycemic patients than in healthy people (25, 26). Although CysC is less affected by age, sex and race, it can also be influenced by diabetes, inflammatory state and abnormal thyroid function (27, 28). Secondly, there is higher proportion of overweight or obese patients in those with diabetes. Muscle mass and diet are different in these patients, leading to bias in them when calculated with eGFR_SCr_ equations (29). Cystatin C can be greatly affected by fat mass, resulting in higher serum level (30, 31). Thirdly, diabetic patients only consisted of <30% of the study population (5, 7, 8) when these equations were developed for eGFR. Moreover, while applying these eGFR equations, accounting for the characteristics of the population is also critical. Thus, the performance of eGFR equations in diabetic population is worse than that in non-diabetic population.

It was obvious that in our study, equations with CysC, including CKD-EPI_CysC_, CAPA, and FAS_CysC_ were more biased than those with SCr in both diabetic and non-diabetic patients. Part of the reasons was the inaccurate standardization method. CysC was measured by nephelometric and turbidimetric methods. Unlike SCr, although standardization was achieved since 2011 with the release of a certified reference material(ERM-DA471/IFCC), which is not as good as IDMS, the “gold standard” of CysC is still lack of reference method comparable to that of SCr (32). Measurements of CysC from different laboratories are still biased (33). A single serum marker may not be able to completely avoid non-GFR factors, and the combination of multiple serum markers may reduce the inaccuracy caused by non-GFR factors (8). Our study showed that combination of SCr and CysC make eGFR more accurate also in diabetic CKD patients.

In the subgroup of diabetic CKD group, CKD-EPI_SCr−CysC_ showed the best performance among CKD-EPI equations, which was similar to Xue's study (34). Previously, Zhao et al. compared FAS equations with CKD-EPI equations and found that the FAS_SCr−CysC_ was better than CKD-EPI_SCr−CysC_ (35). Our data demonstrated that the RLM, FAS_SCr_, and FAS_SCr−CysC_ did not have significantly better performance than CKD-EPI_SCr−CysC_. Since most of the previous studies used renal dynamic imaging as measured GFR, which is thought to be less accurate than iohexol GFR, our study could prove stronger evidence for the result. Further research is needed in the future.

The predictors that might affect the prediction of eGFR are still controversial and researchers have not reached a consensus yet. In addition, understanding the sensitivity and the specificity of eGFR equations while applying them in a particular population is critical (14). Our study further explored the possible factors related to inaccuracy of the eGFR equations in diabetic CKD patients. Our observations show that higher BMI and lower mGFR level were related to less accuracy in CKD-EPI_SCr_ and CKD-EPI_SCr−CysC._ The possible reason is that as BMI increases and mGFR decreases, inflammation and muscle levels in diabetic patients affect levels of serum markers, which are the factors making eGFR inaccurate. Our study also showed that DKD status was an affecting factor related to inaccuracy of three eGFR equations. Patients diagnosed with DKD tend to have a longer course of diabetic disease than CKD patients with diabetes. Their inflammation state, muscle and diet are possible factors infecting eGFR's accuracy. Akihiro Tsuda et al found that eGFR equations developed from Japanese population, were less accurate in diabetic patients (36), and poor glycemic control was a major factor in the overestimation of GFR in patients with hyperglycemia (37). Our study showed poor glucose control (HbA1c ≥ 6.5) was related to inaccuracy in CKD-EPI_SCr_. Furthermore, in a previous study, Masclsaac et al found evidence that in patients <60 years old and whose fasting glucose more than 8 mmol/L, mGFR was higher than eGFR (*P* < 0.01 and *P* < 0.05, respectively) (38). Further researches are needed in the future.

It is important to acknowledge that there were some limitations in this study. Firstly, our patient group was limited by bias toward patients with diabetic CKD, because many of the cases selected were diagnosed with primary glomerular diseases at the same time. Therefore, pure diabetic or diabetic nephropathy patients are likely to be underrepresented. Secondly, our study was cross-sectional and each patient had only one blood draw. In consequence, there were possible systemic errors. Further studies need to be proceeded on more blood draws based on larger sample size. Since eGFR provides unsatisfied accuracy in many situations, mGFR still has its place in clinical practice (39, 40). Hopefully, eGFR equations with different combination of new serum markers could be explored.

In summary, in this cross-sectional study drawn from Chinese diabetic and non-diabetic CKD population, our results provided more evidence to support that CKD-EPI_Scr−CysC_ were more suitable in Chinese diabetic CKD patients. RLM, FAS_SCr_, and FAS_SCr−CysC_ were promising equations. BMI, mGFR, DKD status, and HbA1c were independent factors associated with accuracy in eGFR equations.

## Data Availability

The datasets generated for this study are available on request to the corresponding author.

## Ethics Statement

This study was approved by the Institutional Review Board (IRB) of Ruijin Hospital. Informed consents were signed by patients before recruitment.

## Author Contributions

NC, HS, JX, HR, and WW: design. DX, YD, NC, WZ, HR, LN, BC, HW, and NL: recruitment and data collection. DX, HS, and JX: data analysis. DX, YW, and YL: writing.

### Conflict of Interest Statement

The authors declare that the research was conducted in the absence of any commercial or financial relationships that could be construed as a potential conflict of interest.

## References

[1] Wang L Gao P Zhang M Huang Z Zhang D Deng Q. Prevalence and ethnic pattern of diabetes and prediabetes in China in 2013. JAMA. (2017) 317:2515–23. 10.1001/jama.2017.7596 28655017 PMC5815077

[2] Zhang L Long J Jiang W Shi Y He X Zhou Z. Trends in chronic kidney disease in China. N Eng J Med. (2016) 375:905–6. 10.1056/NEJMc1602469 27579659

[3] Matsushita K van der Velde M Astor BC Woodward M Levey AS de Jong PE. Association of estimated glomerular filtration rate and albuminuria with all-cause and cardiovascular mortality in general population cohorts: a collaborative meta-analysis. Lancet. (2010) 375:2073–81. 10.1016/S0140-6736(10)60674-5 20483451 PMC3993088

[4] van der Velde M Matsushita K Coresh J Astor BC Woodward M Levey A. Lower estimated glomerular filtration rate and higher albuminuria are associated with all-cause and cardiovascular mortality. A collaborative meta-analysis of high-risk population cohorts. Kidney Int. (2011) 79:1341–52. 10.1038/ki.2010.536 21307840

[5] Levey AS Bosch JP Lewis JB Greene T Rogers N Roth D. A more accurate method to estimate glomerular filtration rate from serum creatinine: a new prediction equation. Modification of Diet in Renal Disease Study Group. Ann Intern Med. (1999) 130:461–70. 10.7326/0003-4819-130-6-199903160-00002 10075613

[6] Abbink FCH Laarman CARC Braam KI van Wijk JAE Kors WA Bouman AA Beta-trace protein is not superior to cystatin C for the estimation of GFR in patients receiving corticosteroids. Clin Biochem. (2008) 41:299–305. 10.1016/j.clinbiochem.2007.11.012 18082138

[7] Levey AS Stevens LA Schmid CH Zhang YL Castro AF III Feldman HI. A new equation to estimate glomerular filtration rate. Ann Intern Med. (2009) 150:604–12. 10.7326/0003-4819-150-9-200905050-00006 19414839 PMC2763564

[8] Inker LA Schmid CH Tighiouart H Eckfeldt JH Feldman HI Greene T. Estimating glomerular filtration rate from serum creatinine and cystatin C. N Eng J Med. (2012) 367:20–9. 10.1056/NEJMoa1114248 22762315 PMC4398023

[9] KDIGO Chapter 1: definition and classification of CKD. Kidney Int Suppl. (2013) 3:19–62. 10.1038/kisup.2012.64 PMC4089693 25018975

[10] Nyman U Grubb A Larsson A Hansson LO Flodin M Nordin G. The revised Lund-Malmo GFR estimating equation outperforms MDRD and CKD-EPI across GFR, age and BMI intervals in a large Swedish population. Clin Chem Lab Med. (2014) 52:815–24. 10.1515/cclm-2013-0741 24334413

[11] Grubb A Horio M Hansson LO Bjork J Nyman U Flodin M. Generation of a new cystatin C-based estimating equation for glomerular filtration rate by use of 7 assays standardized to the international calibrator. Clin Chem. (2014) 60:974–86. 10.1373/clinchem.2013.220707 24829272

[12] Pottel H Hoste L Dubourg L Ebert N Schaeffner E Eriksen BO. An estimated glomerular filtration rate equation for the full age spectrum. Nephrol Dial Transpl. (2016) 31:798–806. 10.1093/ndt/gfv454 26932693 PMC4848755

[13] Pottel H Delanaye P Schaeffner E Dubourg L Eriksen BO Melsom T. Estimating glomerular filtration rate for the full age spectrum from serum creatinine and cystatin C. Nephrol Dial Transpl. (2017) 32:497–507. 10.1093/ndt/gfw425 28089986 PMC5837496

[14] Liu X Foster MC Tighiouart H Anderson AH Beck GJ Contreras G. Non-GFR determinants of low-molecular-weight serum protein filtration markers in CKD. Am J Kidney Dis. (2016) 68:892–900. 10.1053/j.ajkd.2016.07.021 27663042 PMC5123901

[15] National Kidney Foundation KDOQI clinical practice guideline for diabetes and CKD: 2012 Update. Am J Kidney Dis. (2012) 60:850–86. 10.1053/j.ajkd.2012.07.005 23067652

[16] Alicic RZ Rooney MT Tuttle KR. Diabetic kidney disease: challenges, progress, and possibilities. Clin J Am Soc Nephrol. (2017) 12:2032–45. 10.2215/CJN.11491116 28522654 PMC5718284

[17] Delanaye P Ebert N Melsom T Gaspari F Mariat C Cavalier E. Iohexol plasma clearance for measuring glomerular filtration rate in clinical practice and research: a review. Part 1: how to measure glomerular filtration rate with iohexol? Clin Kidney J. (2016) 9:682–99. 10.1093/ckj/sfw070 27679715 PMC5036902

[18] Brochner-Mortensen J. A simple method for the determination of glomerular filtration rate. Scand J Clin Lab Investig. (1972) 30:271–4. 10.3109/00365517209084290 4629674

[19] Du Bois D Du Bois EF. A formula to estimate the approximate surface area if height and weight be known. 1916. Nutrition. (1989) 5:303–11. 2520314

[20] Stevens LA Zhang Y Schmid CH. Evaluating the performance of equations for estimating glomerular filtration rate. J Nephrol. (2008) 21:797–807. 19034863 PMC4418188

[21] Foundation NK K/DOQI clinical practice guidelines for chronic kidney disease: evaluation, classification, and stratification. Am J Kidney Dis. (2002) 39:S1–266. 11904577

[22] Nair S Mishra V Hayden K Lisboa PJ Pandya B Vinjamuri S. The four-variable modification of diet in renal disease formula underestimates glomerular filtration rate in obese type 2 diabetic individuals with chronic kidney disease. Diabetologia. (2011) 54:1304–7. 10.1007/s00125-011-2085-9 21359581

[23] Silveiro SP Araujo GN Ferreira MN Souza FD Yamaguchi HM Camargo EG. Chronic Kidney Disease Epidemiology Collaboration (CKD-EPI) equation pronouncedly underestimates glomerular filtration rate in type 2 diabetes. Diabetes Care. (2011) 34:2353–5. 10.2337/dc11-1282 21926286 PMC3198274

[24] Liu X Gan X Chen J Lv L Li M Lou T. A new modified CKD-EPI equation for Chinese patients with type 2 diabetes. PLoS ONE. (2014) 9:e109743. 10.1371/journal.pone.0109743 25313918 PMC4196932

[25] Levey AS Coresh J Greene T Marsh J Stevens LA Kusek JW. Expressing the modification of diet in renal disease study equation for estimating glomerular filtration rate with standardized serum creatinine values. Clin Chem. (2007) 53:766–72. 10.1373/clinchem.2006.077180 17332152

[26] Cheuiche AV Soares AA Camargo EG Weinert LS Camargo JL Silveiro SP. Comparison between IDMS-traceable Jaffe and enzymatic creatinine assays for estimation of glomerular filtration rate by the CKD-EPI equation in healthy and diabetic subjects. Clin Biochem. (2013) 46:1423–9. 10.1016/j.clinbiochem.2013.05.067 23747959

[27] Stevens LA Schmid CH Greene T Li L Beck GJ Joffe MM. Factors other than glomerular filtration rate affect serum cystatin C levels. Kidney Int. (2009) 75:652–60. 10.1038/ki.2008.638 19119287 PMC4557800

[28] Manetti L Pardini E Genovesi M Campomori A Grasso L Morselli LL. Thyroid function differently affects serum cystatin C and creatinine concentrations. J Endocr Investig. (2005) 28:346–9. 10.1007/BF03347201 15966508

[29] Stevens LA Levey AS. Use of the MDRD study equation to estimate kidney function for drug dosing. Clin Pharmacol Ther. (2009) 86:465–7. 10.1038/clpt.2009.124 19844220

[30] Knight EL Verhave JC Spiegelman D Hillege HL de Zeeuw D Curhan GC. Factors influencing serum cystatin C levels other than renal function and the impact on renal function measurement. Kidney Int. (2004) 65:1416–21. 10.1111/j.1523-1755.2004.00517.x 15086483

[31] Vupputuri S Fox CS Coresh J Woodward M Muntner P. Differential estimation of CKD using creatinine- versus cystatin C-based estimating equations by category of body mass index. Am J Kidney Dis. (2009) 53:993–1001. 10.1053/j.ajkd.2008.12.043 19394726 PMC3028436

[32] Delanaye P Cavalier E Cristol JP Delanghe JR. Calibration and precision of serum creatinine and plasma cystatin C measurement: impact on the estimation of glomerular filtration rate. J Nephrol. (2014) 27:467–75. 10.1007/s40620-014-0087-7 24711159

[33] Bargnoux AS Pieroni L Cristol JP Kuster N Delanaye P Carlier MC. Multicenter evaluation of cystatin c measurement after assay standardization. Clin Chem. (2017) 63:833–41. 10.1373/clinchem.2016.264325 28188233

[34] Chi XH Li GP Wang QS Qi YS Huang K Zhang Q. CKD-EPI creatinine-cystatin C glomerular filtration rate estimation equation seems more suitable for Chinese patients with chronic kidney disease than other equations. BMC Nephrol. (2017) 18:226. 10.1186/s12882-017-0637-z 28693441 PMC5504640

[35] Yong Z Li F Pei X Liu X Song D Zhang X. A comparison between 2017 FAS and 2012 CKD-EPI equations: a multi-center validation study in Chinese adult population. Int Urol Nephrol. (2019) 51:139–46. 10.1007/s11255-018-1997-4 30357600

[36] Tsuda A Ishimura E Uedono H Yasumoto M Ichii M Nakatani S. Comparison of the estimated Glomerular Filtration Rate (eGFR) in diabetic patients, non-diabetic patients and living kidney donors. Kidney Blood Pressure Res. (2016) 41:40–7. 10.1159/000368545 26836393

[37] Tsuda A Ishimura E Ohno Y Ichii M Nakatani S Machida Y Poor glycemic control is a major factor in the overestimation of glomerular filtration rate in diabetic patients. Diabetes Care. (2014) 37:596–603. 10.2337/dc13-1899 24130341

[38] MacIsaac RJ Ekinci EI Premaratne E Lu ZX Seah JM Li Y The Chronic Kidney Disease-Epidemiology Collaboration (CKD-EPI) equation does not improve the underestimation of Glomerular Filtration Rate (GFR) in people with diabetes and preserved renal function. BMC Nephrol. (2015) 16:198 10.1186/s12882-015-0196-0 26630928 PMC4668645

[39] Luis-Lima S Escamilla-Cabrera B Negrin-Mena N Estupinan S Delgado-Mallen P Marrero-Miranda D. Chronic kidney disease staging with cystatin C or creatinine-based formulas: flipping the coin. Nephrology Dial Transpl. (2019) 34:287–94. 10.1093/ndt/gfy086 29762739

[40] Agarwal R Delanaye P. Glomerular filtration rate: when to measure and in which patients? Nephrol Dial Transpl. (2018). [Epub ahead of print]. 10.1093/ndt/gfy363 30520986

