# Supplementary material for: A Validation Study on eGFR Equations in Chinese Patients With Diabetic or Non-diabetic CKD
Source: Front Endocrinol (Lausanne). 2019 Aug 26;10:581. doi: 10.3389/fendo.2019.00581 (PMC6718123; doi:10.3389/fendo.2019.00581)
Supplement: Supplementary file 1 [file Data_Sheet_1.docx]

Supplemental Figure 1:a comparison of mGFRs.


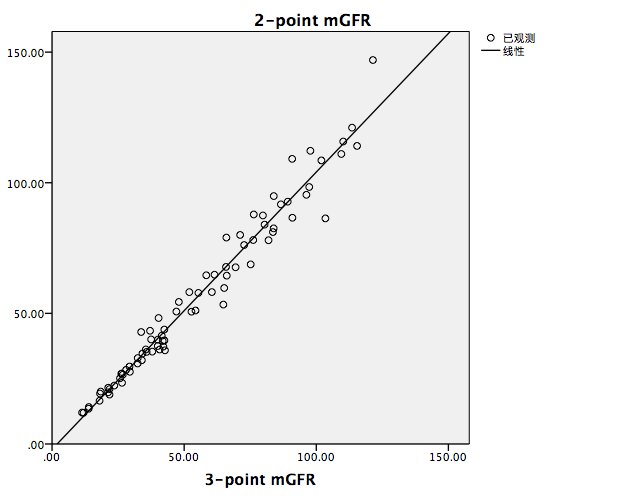


Two types of mGFRs were compared in 80 patients who were taken 3-point blood draws (30 patients were diabetic CKD patients). Among them, the first and third points blood samples were used to calculate the 2-point mGFRs. By correlation analysis, a spline fit curve was established which showed good consistency. R^2^: 0.966; Slope: 1.061(P<0.001); Intercept: -2.048(P=0.142).

Supplemental Table 1: eGFR equations included in this study.

| CKD-EPI_SCr_  = 141 × min (SCr/κ, 1)α× max (SCr/κ, 1)–1.209 × 0.993age (years) (×1.018 if female)  (κ is 0.7 for females and 0.9 for males; α is –0.329 for females and –0.411 for males) |
| --- |
| CKD-EPI_CysC_  =133 × min (CysC/0.8, 1)–0.499 × max (CysC/0.8, 1)–1.328 × 0.996age (years) (×0.932 if female) |
| CKD-EPI_SCr-CysC_  = 135 × min (SCr/κ, 1)α× max (SCr/κ, 1)–0.601 × min (CysC/0.8, 1)–0.375 × max (CysC/0.8, 1)–0.711 × 0.995age (years) (×0.969 if female) |
| (κ is 0.7 for females and 0.9 for males; α is –0.248 for females and –0.207 for males.) |
| Revised Lund-Malmö Study equation (RLM) |
| =e^X-0.0158×Age+0.438×ln(Age)^ |
| Female pCr＜150μmol/L：X=2.50+0.0121×(150-pCr)  Female pCr≥150μmol/L：X=2.50-0.926×ln(pCr/150) |
| Male pCr＜180μmol/L：X=2.56+0.00968×(180-pCr) |
| Male pCr≥180μmol/L：X=2.56-0.926×ln(pCr/180) |
| CAPA equation  = 130 × CysC^-1.069^× age^-0.117^ - 7 |
| FAS_SCr_  = 107.3/(SCr/Q_SCr_) ×[ 0.988^(Age-40)^when age＞40 years]  Q-values are the mean or median SCr value for age-/sex-specific healthy populations. |
| FAS_CysC_  =107.3/(CysC/Q_CysC_) × [ 0.988^(Age-40)^ when age＞40 years ] |
| FAS_SCr-CysC_  = 107.3/[α× (SCr/Q_SCr_) +(1-α)×(CysC/Q_CysC_) ] × [ 0.988^(Age-40)^ when age＞40 years ] |

The unit for SCr is mg/dl in CKD-EPI equations and mmol/L in FAS equations.

Supplemental Table 2: Bias, precision and accuracy of different equations in CKD patients without DM

|  | Bias | | Precision | Accuracy | |
| --- | --- | --- | --- | --- | --- |
|  | MD | AMD | IR | P30 | P10 |
| CKD-EPI_SCr_ | -1.48(-2.57,-0.60) | 6.28(4.89,8.25) | 12.08(8.29,15.94) | 75.52(69.03,81.09) | 40.63(32.16,46.27) |
| CKD-EPI_CysC_ | -7.39(-10.04,-5.49) | 8.84(7.67,11.04) | 13.83(10.76,16.19) | 63.02(55.99,68.87) | 23.44(16.58,28.36) |
| CKD-EPI_SCr-CysC_ | -5.28(-6.52,-4.09) | 6.67(5.54,7.70) | 10.88(8.33,13.65) | 77.60(71.53,83.32) | 31.77(24.33,37.12) |
| RLM | -3.18 (-4.20, -2.05) | 6.58 (5.07, 7.55) | 14.24 (10.90, 17.58) | 77.08(70.43,82.68) | 30.21 (23.02, 36.23) |
| CAPA | -8.06 (-9.84, -6.63) | 9.57 (7.99,11.04) | 13.83 (11.69, 15.87) | 64.58 (56.82, 70.29) | 22.40 (15.61, 27.36) |
| FAS_SCr_ | 1.30 (-0.74, 2.50) ^a^ | 7.35 (6.53, 8.77) | 14.63 (11.15, 17.57) | 80.21 (74.71, 85.76) | 30.73 (21.80, 34.60) |
| FAS_CysC_ | -2.92 (-5.34, -1.43) | 8.06 (6.01, 9.53) | 19.41 (16.34, 22.67) | 71.35 (63.80, 76.61) | 28.13 (21.18, 33.86) |
| FAS_SCr-CysC_ | -1.19 (-2.49, 0.15) | 6.44 (5.00, 7.59) | 13.36 (10.11, 15.98) | 82.29 (76.21, 87.38) | 36.46 (28.48, 42.23) |

MD, median difference; AMD, absolute median difference; IR, interquartile range. Data were showed as value (95% confidence interval, 95% CI).CIs for the metrics were calculated by means of bootstrap methods(1000 bootstraps). Unbiased results are ^a^. RLM, Revised Lund-Malmö equation.FAS, Full Age Spectrum equation.

Supplemental Table 3:Clinical factors related to the accuracy of eGFR.

| Variables | Overall  (N) | CKD-EPI_Scr_ | | |  | CKD-EPI_CysC_ | |  |  |  | CKD-EPI_SCr-CysC_ | |
| --- | --- | --- | --- | --- | --- | --- | --- | --- | --- | --- | --- | --- |
|  |  | D<30%  (n) | D<30% rate (%) | P |  | D<30%  (n) | D<30% rate (%) | P |  | D<30%  (n) | D<30% rate (%) | P |
| Total | 180 | 127 | 70.1 |  |  | 106 | 58.9 |  |  | 123 | 68.3 |  |
| Age (year) |  |  |  | 0.939 |  |  |  | 0.783 |  |  |  | 0.957 |
| <65 | 123 | 87 | 70.7 |  |  | 72 | 58.5 |  |  | 88 | 71.5 |  |
| ≥65 | 57 | 40 | 70.2 |  |  | 34 | 59.6 |  |  | 41 | 71.9 |  |
| Gender |  |  |  | 0.133 |  |  |  | 0.592 |  |  |  | 0.726 |
| male | 120 | 89 | 74.2 |  |  | 69 | 57.5 |  |  | 87 | 72.5 |  |
| female | 60 | 38 | 63.3 |  |  | 37 | 61.7 |  |  | 42 | 70.0 |  |
| BMI |  |  |  | 0.668 |  |  |  | 0.324 |  |  |  | 0.296 |
| <24 | 65 | 48 | 73.8 |  |  | 43 | 66.2 |  |  | 49 | 75.4 |  |
| 24~28 | 63 | 42 | 66.7 |  |  | 34 | 54.0 |  |  | 47 | 74.6 |  |
| ≥28 | 52 | 37 | 71.2 |  |  | 29 | 55.8 |  |  | 33 | 63.5 |  |
| mGFR |  |  |  | <0.001 |  |  |  | 0.100 |  |  |  | 0.020 |
| ≥60 | 60 | 51 | 85.0 |  |  | 42 | 70.0 |  |  | 50 | 83.3 |  |
| 30-60 | 68 | 52 | 76.5 |  |  | 36 | 52.9 |  |  | 48 | 70.6 |  |
| <30 | 52 | 24 | 46.2 |  |  | 28 | 53.8 |  |  | 31 | 59.6 |  |
| DKD status |  |  |  | 0.281 |  |  |  | 0.072 |  |  |  | 0.043 |
| DKD | 64 | 42 | 65.6 |  |  | 32 | 50.0 |  |  | 40 | 62.5 |  |
| Non-DKD | 116 | 85 | 73.3 |  |  | 74 | 63.8 |  |  | 89 | 76.7 |  |
| HbA1c |  |  |  | 0.331 |  |  |  | 0.953 |  |  |  | 0.292 |
| <6.5 | 71 | 53 | 74.6 |  |  | 42 | 59.2 |  |  | 54 | 76.1 |  |
| ≥6.5 | 109 | 74 | 67.9 |  |  | 64 | 58.7 |  |  | 75 | 68.8 |  |

BMI: body mass index; DKD: diabetic kidney disease; mGFR: measured glomerular filtration rate; N, numbers; HbA1c: glycated hemoglobin.
